# A Picosecond Delay Generator Optimized by Layout and Routing Based on FPGA

**DOI:** 10.3390/s23136144

**Published:** 2023-07-04

**Authors:** Min Zhu, Tang Cui, Xihan Qi, Qiang Gao

**Affiliations:** 1 School of Electrical Engineering and Automation, Harbin Institute of Technology, Harbin 150001, China; zhumin@hit.edu.cn (M.Z.); 22s006055@stu.hit.edu.cn (X.Q.); 2 Chongqing Research Institute of HIT, Chongqing 401151, China; 3 School of Mechatronics Engineering, Harbin Institute of Technology, Harbin 150001, China; gaoq@hit.edu.cn

**Keywords:** CARRY4, carry delay chain, delay generator, FPGA

## Abstract

**Featured Application:**

**A delay generator can be used for signal synchronization. Generally, there is a relative delay between the external trigger signal and the reference. The delay generator can delay the external trigger signal to greatly reduce the relative delay between the external trigger signal and the reference, so as to achieve the effect of signal synchronization.**

**Abstract:**

A delay generator is a timing control device that can generate a delay for the input signal according to the actual requirements. A delay generator with a combination of rough delay and precise delay is implemented on a Xilinx Kintex-7 series FPGA with a design scheme based on carry delay chain. The delay generator uses the delay time parameters sent by the upper monitor to work and to reflect the current working state to the upper monitor. In this article, a theoretical model of the delay generator is designed, and a delay compensation scheme is proposed to make the working state of the theoretical model closer to the actual circuit. Through simulation experiments, the time resolution of the delay generator is 54 ps, and the time accuracy is less than 50 ps. The delay scheme adopted in this article is highly scalable, and the time resolution and time accuracy can be further improved. Finally, a theoretical model of the delay generator with relatively high time resolution is implemented through low resource occupancy rate and little workload.

## 1. Introduction

**Figure 1:**
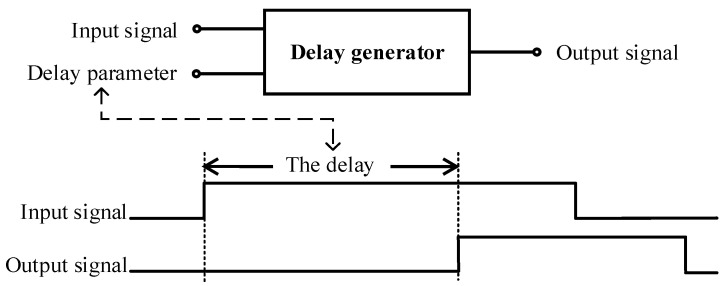
The main function diagram of the delay generator.

The delay generator [1,2], also known as a synchronization device, is a timing control device. In simple terms, the main function of this device is to delay a signal accordingly, that is, to delay the external trigger signal, and its main function is shown in Figure 1. It is widely used in the fields of laser radar [3], nuclear physics [4], automated testing equipment [5], medical imaging [6], and spectral instruments [7]. For example, in many synchronous communication systems, digital signals need to be accurately delayed to achieve signal synchronization [8]. In the ultra-high-speed framing camera [9], it is necessary to accurately delay the control signal to achieve the effect that the time interval between each shutter opening is strictly equal, so that the ultra-high-speed framing camera can work stably.

In addition to the fields mentioned above, the delay generator can also be used in industrial fields, such as multi-phase, high-precision power supplies and multi-axis robotic arms. The multi-phase high-precision power supply needs to strictly control the timing between phases during the working process, and the delay generator can well adjust the timing of the control signal. The multi-axis robotic arms need to coordinate the motion of each axis during the working process, and the delay generator can reduce the inconsistency caused by the control error. In fact, delay generators have a large number of applications in many fields that require the precise control of signal timing. 

The focus of the delay generator designed in this article is a time method (structure) called a carry delay chain [10,11,12], which is mainly used in various delay systems and time interval measurement systems, and it is currently used more frequently as a time method. In the field of time interval measurement, this time method is also called delay line interpolation [13,14,15]. The carry delay chain is mainly implemented in a FPGA or an ASIC, which have high degrees of integration. This article will use FPGA to implement the delay generator. 

In 2011, Yan Song et al. [16] described a novel FPGA-based digital delay generator (DDG). This DDG also adopts the scheme of combining rough delay and precise delay, and it uses the AD9501 chip to compensate the time interval. This DDG had a delay range of 4.4 ms and a time resolution of 65 ps. This article presents a novel architecture of FPGA combined with a AD9501 chip, but the complexity of the architecture reduces the reliability of its work. 

In 2015, Shadi MS Harb et al. [17] described a new architecture of an on-chip multilateral delay generator with high time resolution based on Vernier technology, which utilizes multiple edge-triggered oscillators to achieve high time resolution. Simulation results show that the delay resolution can reach 14.6 ps when the reference system clock frequency is 500 MHz, which is based on 0.09 μm CMOS technology. Compared with the previous technology, the multi-VDL structure further improves the time resolution and the delay accuracy. 

In 2019, Ke Cui et al. [18] described a FPGA-based high-resolution Vernier delay generator that adopts a novel delay chain structure based on multiplexed carry chains. A multiplexer placed next to the carry chain allows the actual delay of the delay chain to be dynamically adjusted while maintaining a high resolution. The delay generator prototype circuit is implemented on Stratix III FPGA with an experimental time resolution of 23.9 ps, and it has a high linearity. 

In this article, a novel delay generator architecture, based on a carry delay chain, is proposed to generate high precision delay, and it is deployed in a Xilinx Kintex-7 series FPGA. The upper monitor sends configuration parameters and instructions to the FPGA through the serial port to control its working state. The upper monitor used in this article is a general personal computer. The delay generator designed in this article refers to the related technology of time interval measurement, but its main function is time interval generation. Compared with the previous technology [17], the difference in the delay generator designed in this article is that it not only needs to generate time interval, but it also has real-time requirements, that is, it needs to delay the random external trigger signal at the arrival time. 

## 2. Related Principles and Circuits

### 2.1. The Principle of the Carry Delay Chain

**Figure 2:**
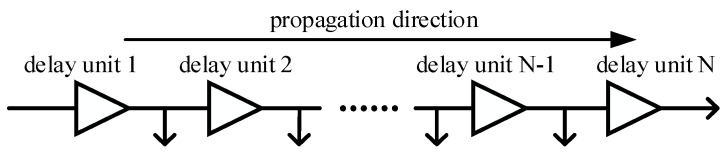
The carry delay chain structure diagram.

**Figure 3:**
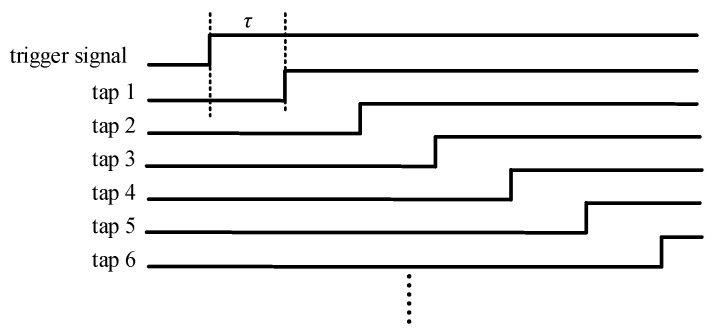
The tap signal waveform.

The carry delay chain [10,11,12] is a circuit structure, which is composed of a number of basic delay units. Its structure is shown in Figure 2. Each delay unit can generate delay time, and delay units are generally implemented by specific hardware circuits. A tap needs to be introduced between every two adjacent delay units to check the current working status of the delay chain. After the external trigger signal enters the delay chain and passes through each delay unit in turn, the “staircase” waveform shown in Figure 3 will be obtained. 

By recording the signal at the tap of the carry delay chain, the actual number of delay units passed by the external trigger signal can be obtained. If the delay time generated by each delay unit is *τ*, the time interval between the external trigger signal and the stop signal is approximately the number of delay units through which the external trigger signal passes multiplied by *τ*. Obviously, the smaller the *τ*, the higher the time resolution. 

This article produces precise delays by controlling the carry delay chain. In the process of chip manufacturing, the delay time of each delay unit cannot be completely consistent, showing a nonlinearity. In addition, factors such as temperature and voltage of the delay unit will also cause unpredictable errors. However, the delay generator designed in this article is only a theoretical model, without considering the above factors. 

### 2.2. The Implementation of the Carry Delay Chain

**Figure 4:**
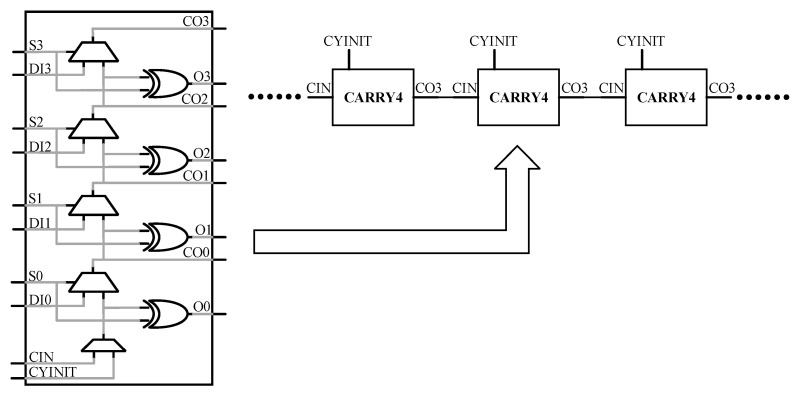
The structure of the CARRY4 unit and the carry delay chain.

This article uses a Xilinx Kintex-7 series FPGA to implement the carry delay chain, whose model is XC7K325TFFG676-2. The dedicated carry chain primitive CARRY4 [19], provided by Xilinx, is used to instantiate the carry delay chain without customizing the IP cores, whose structure is shown in Figure 4. 

The CARRY4 unit consists of four data selectors, four XOR gates, and one OR gate, which can lead to four taps (CO0, CO1, CO2, and CO3). Since the carry-ahead characteristic of the CARRY4 unit will cause confusion in the timing [20], generally the CARRY4 unit will not lead out all four taps, and only one tap CO3 is introduced in this article. Two taps of the CARRY4 unit, CIN and CO3, are used to build the carry delay chain, and the CO3 tap of the previous CARRY4 unit is connected to the CIN tap of the next CARRY4 unit. In this article, a CARRY4 unit is used as a delay unit. 

It can be measured by simulation that each delay unit in the carry delay chain can generate a delay of 54 ps. The carry delay chain has high time resolution, but the delay time generated by each delay unit is very short, so the total delay time generated by a carry delay chain is also very short. Therefore, in this article, the scheme of combining rough delay and precise delay [21,22] will be used to generate a long delay, while, at the same time, ensuring a high time resolution. 

## 3. The Implementation of the Delay Scheme

**Figure 5:**
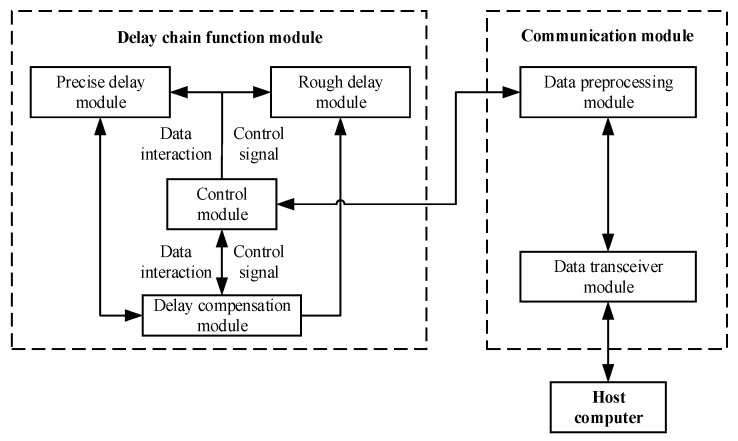
The structure diagram of the whole device.

The delay generator delays the external trigger signal according to the delay time parameters sent by the upper monitor. The structure of the delay generator is shown in Figure 5, and the whole device consists of two modules: the delay chain function module and the communication module. The delay chain function module is the core module of this article, which is used to generate the delay. The communication module communicates with the upper monitor, receives the delay time parameters sent by the upper monitor, and reflects the working status of the delay generator to the upper monitor.

**Figure 6:**
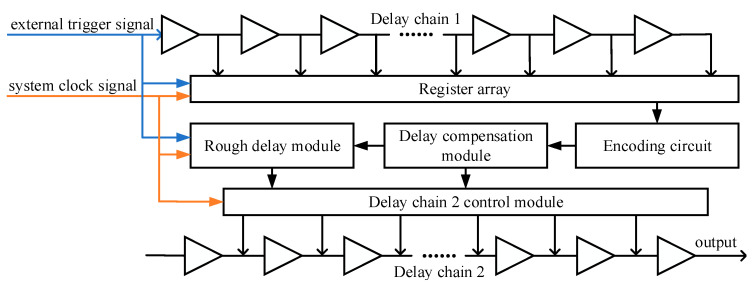
The structure diagram of the delay chain function module.

**Figure 7:**
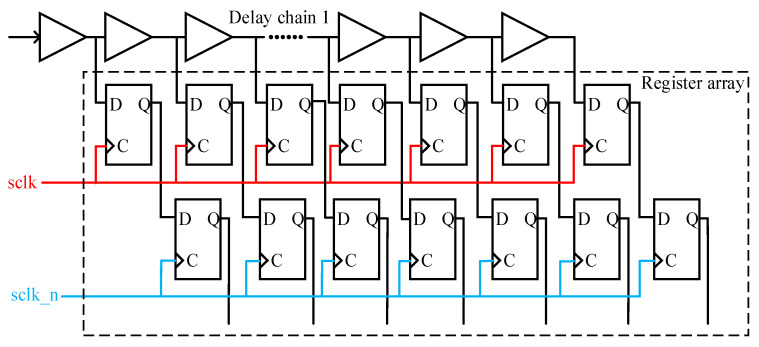
The structure diagram of the register array.

The delay chain function module is used to realize the main function of the delay generator, and its structure is shown in Figure 6. The system clock frequency used in this article is 100 MHZ, and both delay chain 1 and delay chain 2 are constructed by 250 CARRY4 units. The register array is used to save the measurement results of delay chain 1, and the encoding circuit encodes the measurement results of delay chain 1 into binary numbers for the calculation of subsequent circuits. The structure of the register array is shown in Figure 7, which consists of two levels of registers. The two-level registers can avoid metastability affecting the subsequent circuit, where sclk and sclk_n are system clock signals, and the phase difference between the two system clock signals is exactly 180 degrees, that is, the sampling time interval between the two-level registers is 5 ns. The function of the delay chain 2 control module is mainly divided into two parts. Firstly, the input data of delay chain 2 is decoded, and then the decoded data is saved using a register array. The input data is not input from the start point of delay chain 2, as shown in Figure 4, and the input data of delay chain 2 is input into delay chain 2 from the CYINIT port of the CARRY4 unit. 

**Figure 8:**
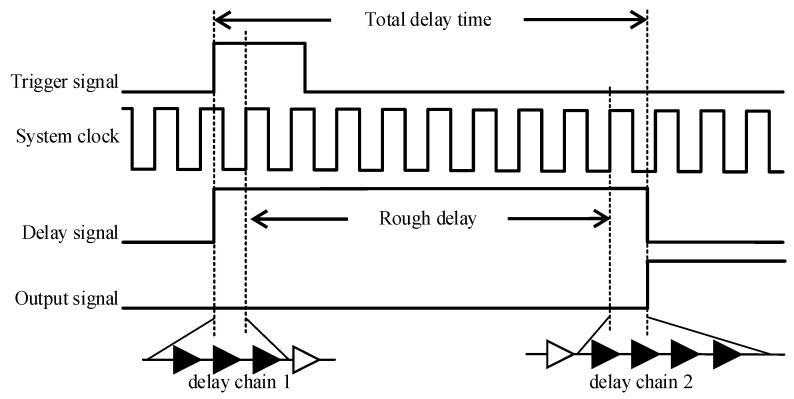
The overall working principle diagram.

Rough delay is to use a general counter to generate a delay longer than one system clock cycle, and precise delay is to use the carry delay chain constructed above to generate a delay shorter than one system clock cycle. The overall principle of this scheme is shown in Figure 8. 

When the external trigger signal arrives, there will be a time interval between the rising edge of the external trigger signal and the rising edge of the system clock signal, which is shorter than one system clock cycle, as shown in Figure 8. Delay chain 1 is used to measure this time interval [23]. When the next rising edge of the system clock signal arrives, the measurement result of delay chain 1 is read, and the rough delay module is started. Then, according to the delay time parameters received by the communication module, the measurement result of the delay chain 1 is subtracted from the preset delay to obtain the remaining delay. All calculations in this scheme use single-precision floating-point numbers. The rough delay module and delay chain 2 are used to generate the remaining delay mentioned above.

**Figure 9:**
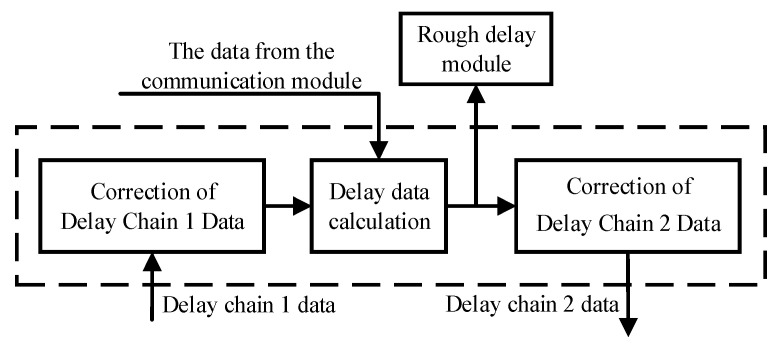
The function of the delay compensation module.

The delay compensation module is the core component of the delay chain function module, and its function is shown in Figure 9. It is mainly used to correct the measurement results of delay chain 1 and the input data of delay chain 2, so that the working state of the theoretical model of delay generator is closer to the actual circuit. The detailed functions of this module will be introduced in the next section. 

## 4. Placement Design and Correction Work

### 4.1. Carry Delay Chain Placement Design

**Figure 10:**
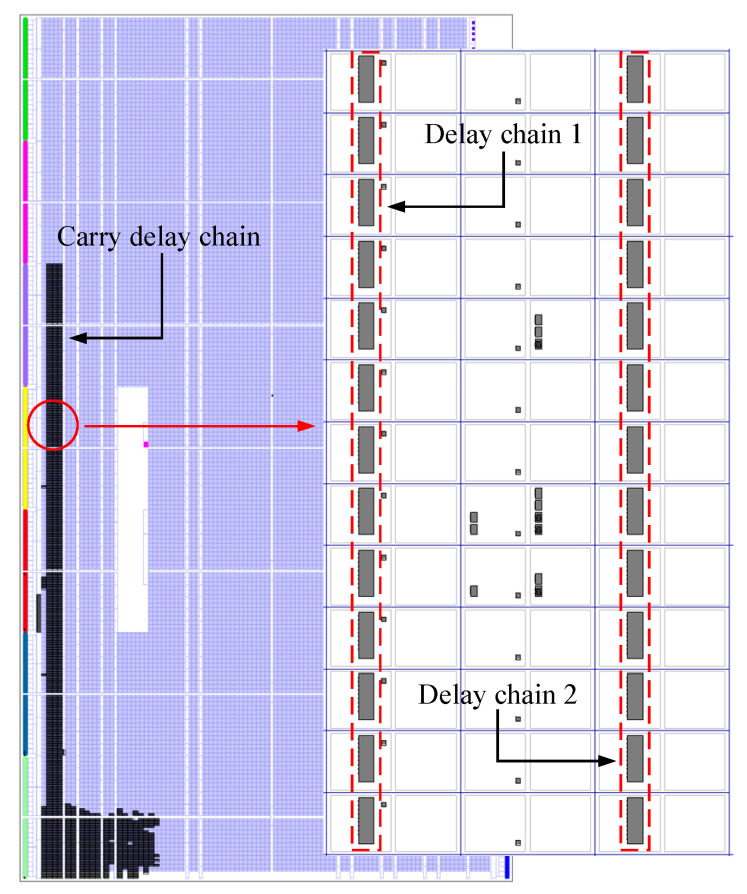
The placement of delay chain 1 and delay chain 2.

The placements of delay chain 1 and delay chain 2 are very important, which will directly affect the time resolution of the entire device. Theoretically, the precise delay is mainly generated by the delay unit, but, in fact, in the FPGA, in addition to the logic unit, the physical wiring also generates delay. Additionally, because the time resolution required in this article is very high, the delay generated by the physical wiring cannot be ignored. Therefore, the delay unit and the physical wiring connected to it should be considered as a whole. To ensure that the delay chain has a high linearity, the physical wiring between two adjacent delay units should be strictly equal in length. To achieve this effect, specific physical constraints on the delay units are required so that they are evenly distributed in the FPGA, as shown in Figure 10.

**Figure 11:**
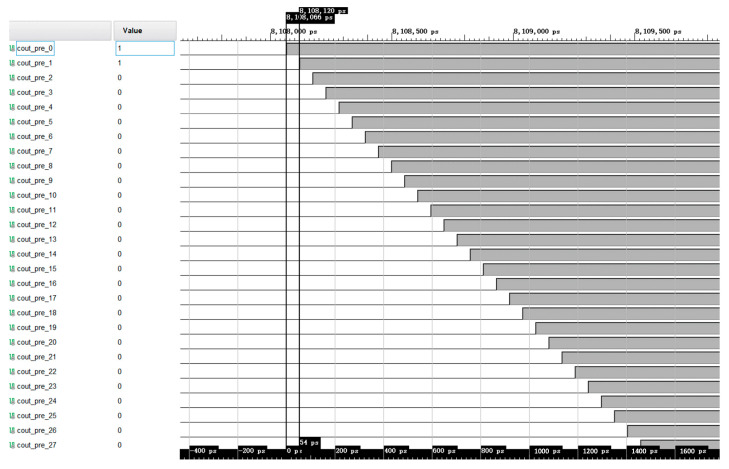
The timing simulation waveform of the carry delay chain.

Delay chain 1 and delay chain 2 not only need to be evenly distributed in the FPGA, but they also need to ensure a high degree of consistency, so two specific columns of CARRY4 units are selected to construct the delay chain. After timing simulation verification, the delay time generated by each delay unit of delay chain 1 and delay chain 2 is 54 ps, and the timing simulation results are shown in Figure 11. 

### 4.2. Delay Chain 1 Measurement Results Correction

The correction of the measurement results of delay chain 1 is part of the function of the delay compensation module. Due to wiring delay, logic cell delay, clock skew, and other factors, there is an error between the measurement results of delay chain 1 and the actual value, so it is necessary to correct the measurement results of delay chain 1.

**Figure 12:**
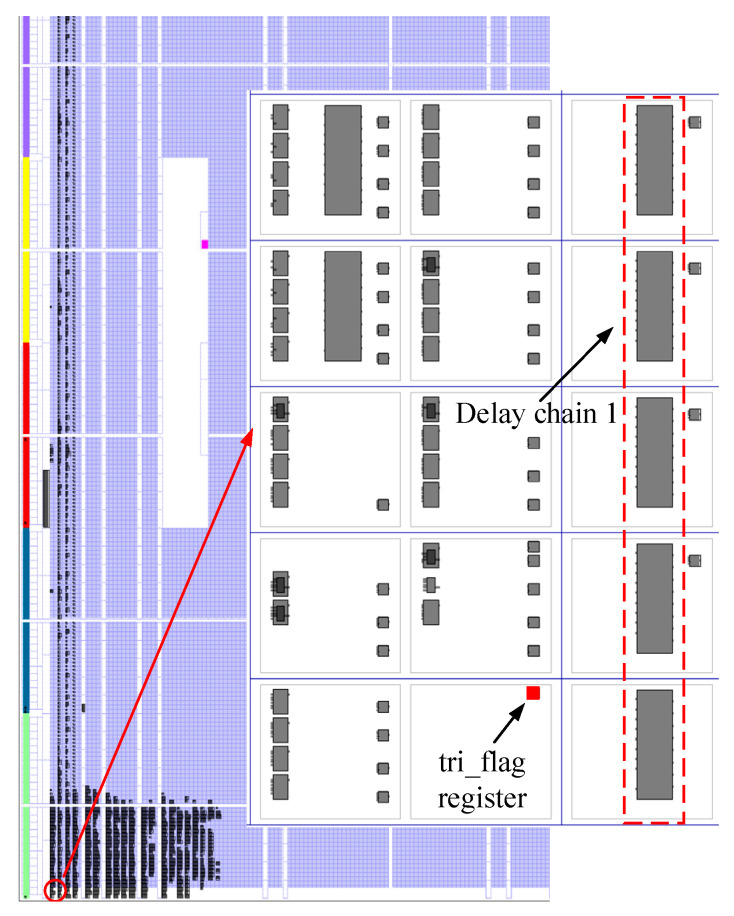
Delay chain 1 and the related hardware resource placement.

**Figure 13:**
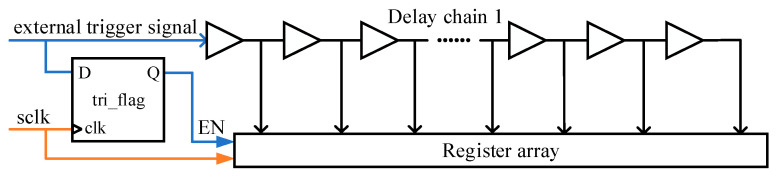
A diagram of the input and output of the tri_flag register.

Before the correction work, the placement of the tri_flag register needs to be fixed, as shown in Figure 12, because the location of the tri_flag register will affect the results of the correction. The tri_flag register is fixed near delay chain 1. The tri_flag register is used to control the sampling of the register array, and its input and output are shown in Figure 13. The input of the tri_flag register is the external trigger signal, and the output is the enable signal of the register array.

**Figure 14:**
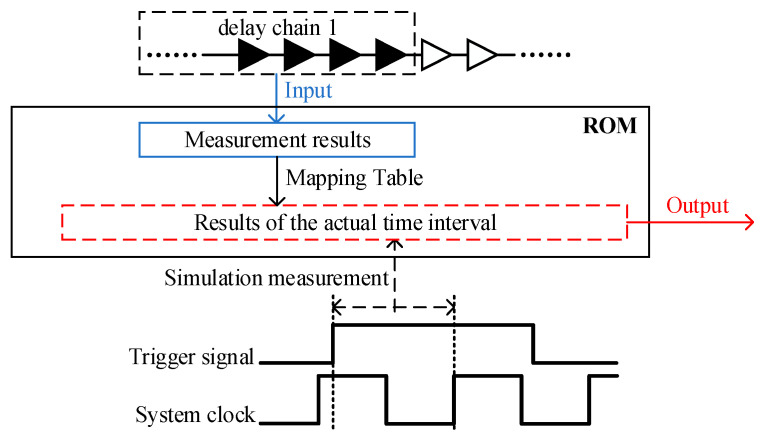
Diagram of the correction of the measurement results of delay chain 1.

After the placement of the tri_flag register is fixed, the measurement results of delay chain 1 can be corrected. The point-by-point correction method is used to correct the measurement results of delay chain 1. The number of measurement results of delay chain 1 is limited, and the number of corresponding correction values is also limited. In timing simulation, all the measurement results are found by changing the arrival time of the external trigger signal constantly, and then they are corrected, and, finally, they form a mapping table, as shown in Figure 14. The mapping table can be implemented by ROM. The measurement results of delay chain 1 directly correspond to the actual time interval.

### 4.3. Correction of Input Data for Delay Chain 2

**Figure 15:**
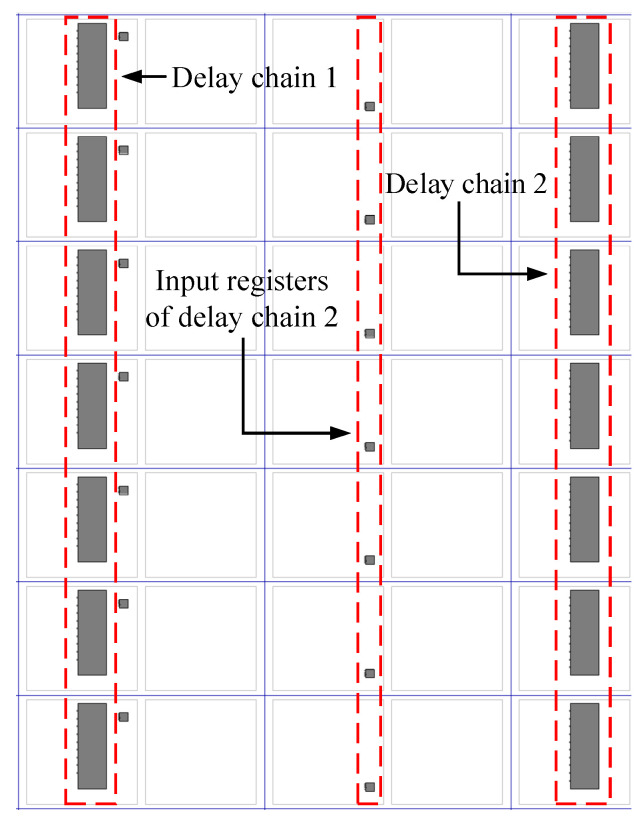
Placement of delay chain 2 and the input registers.

The input data of delay chain 2 are controlled by a group of registers. When the system clock signal arrives, the registers will output the saved data to delay chain 2. In order to ensure the time accuracy of the delay scheme, the wiring length between each register mentioned above and delay chain 2 should be strictly equal, as shown in Figure 15. The input registers of delay chain 2 are also evenly distributed in the FPGA.

**Figure 16:**
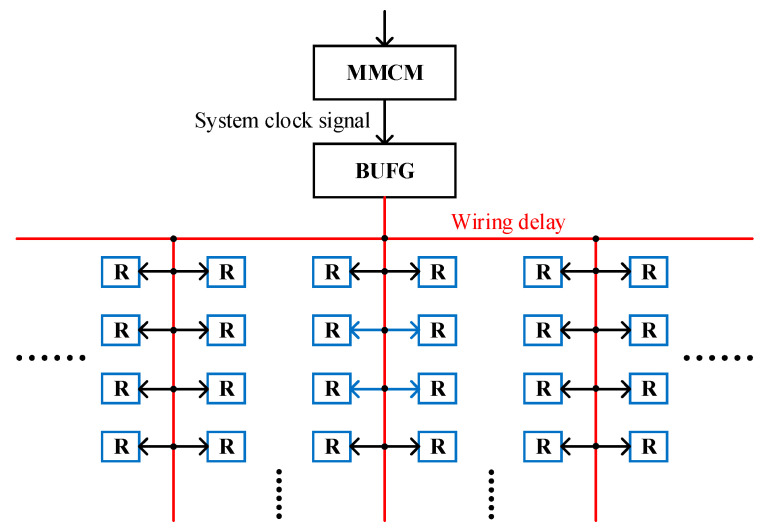
A structure diagram of system clock of the FPGA.

In the FPGA, the mixed-mode clock manager (MMCM) generates the system clock signal, which is then input to the global buffer (BUFG), and finally the system clock signal is output from BUFG to each register, as shown in Figure 16. However, due to the wiring delay, it is impossible for the system clock signal to reach each register at the same time, so it is necessary to minimize the error caused by wiring delay as much as possible. 

**Figure 17:**
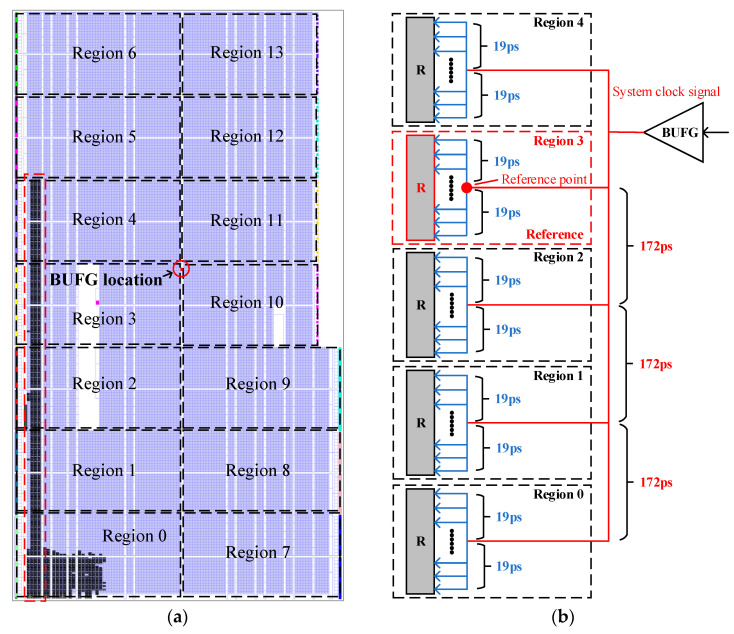
(**a**) FPGA hardware resource distribution. (**b**) System clock signal wire placement diagram.

The hardware resources of the FPGA are divided into several clock regions, as shown in Figure 17a. As mentioned above, the system clock signal is output from the BUFG to each register, and each clock region leads a wire from the BUFG to provide the system clock signal for this region, as shown in Figure 17b. 

The relative delay between the system clock signals input to each register in the same clock region is very small, and it is measured at a maximum of 19 ps through timing simulation, as shown in Figure 17b. The relative delay between the system clock signals input to different clock regions is large, and the relative delay between the system clock signals input to adjacent clock regions is 172 ps, which is measured by timing simulation, as shown in Figure 17b. 

Since the wiring delay is unavoidable, then the wiring delay can be counted as part of the overall delay, so a reference is needed as a standard for calculation. In Figure 17a, the carry delay chains are evenly distributed among five clock regions, and the BUFG is located between region 3 and 4, so region 3 is chosen as a reference, as shown in Figure 17b. Then, based on the reference, the relative delays of all registers can be obtained.

**Figure 18:**
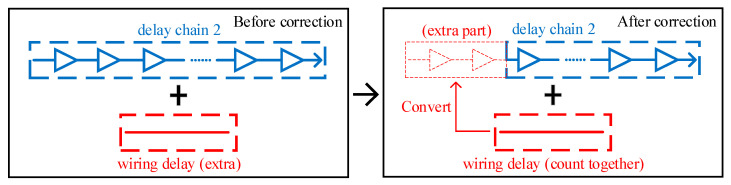
A diagram of the input data correction of delay chain 2.

The correction of the input data of delay chain 2 is part of the function of the delay compensation module, and its principle is shown in Figure 18. Before correction, the wiring delay is extra to the overall delay, in order to calculate the wiring delay as part of the overall delay, and the input data of delay chain 2 need to be changed. After correction, the relative delay of the system clock signal mentioned above is subtracted from the input data of delay chain 2 so as to shorten the delay generated by delay chain 2. That is, the extra wiring delay is counted as part of the overall delay.

## 5. Results

In this section, the delay generator is tested through a large number of simulation experiments to verify whether its performance can meet the requirements. Since there are too many experimental data, only two groups of typical experimental data are given here to illustrate the conclusion, and each group of experiments contains 50 data. 

**Figure 19:**
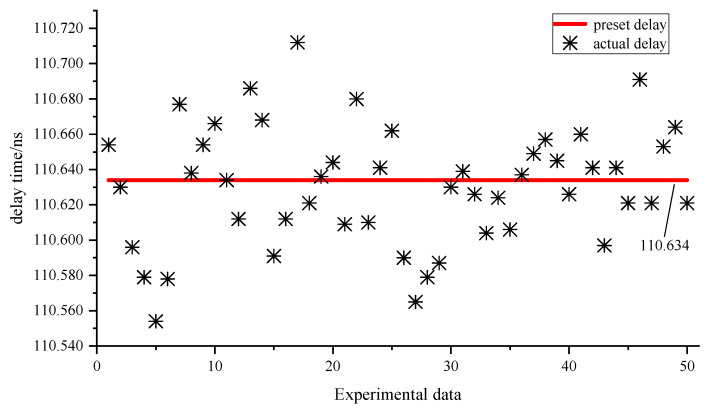
The first group of experimental data.

The preset delay in the first group of experiments is 110.634 ns. In timing simulation, the timing of the external trigger signal is changed by changing the excitation signal in the testbench file, thus changing the offsets relative to the system clock edge, so that the external trigger signal edge is randomly distributed in a system clock cycle. By changing the excitation signal in the testbench file, the external trigger signal randomly generates 50 times of rising edge, and then the cursor function in the simulation is used to measure the time interval between the external trigger signal and the output signal, so as to obtain 50 experimental data, as shown in Figure 19 and Table 1. The maximum error between the preset delay and the 95% confidence interval is less than two times the time resolution, while the standard deviation is less than 50 ps.

**Figure 20:**
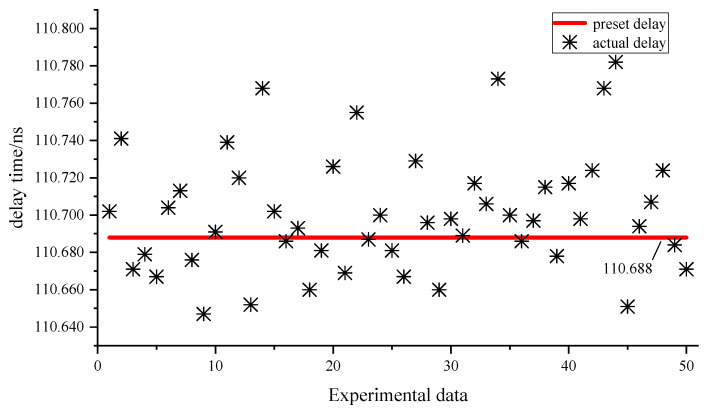
The second group of experimental data.

Next, in the second group of experiments, change the preset delay to 110.688 ns, and repeat the operation of the first group of experiments, as shown in Figure 20 and Table 2. The maximum error between the preset delay and the 95% confidence interval is less than two times the time resolution, while the standard deviation is less than 50 ps.

**Figure 21:**
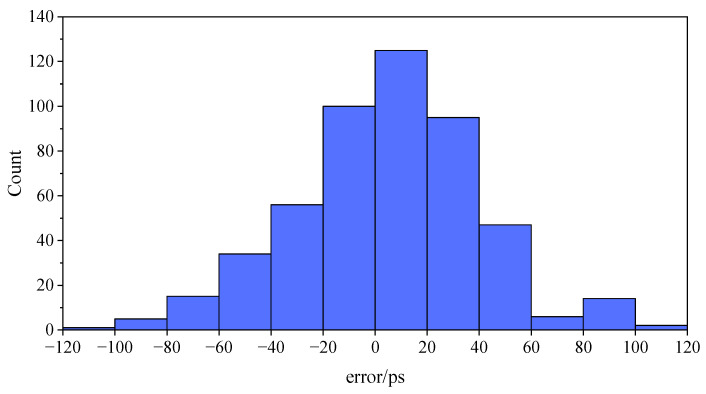
An error histogram of the experimental data.

The preset delay is constantly changed, and the above operation is repeated to obtain 10 groups of experimental data. Statistics and analysis are carried out on the errors of the 10 groups of experimental data, as shown in Figure 21 and Table 3, where the preset delay ranges from 100 to 120 ns. Obviously, the error histogram is close to that of the standard form, and the main distribution of the error ranges from −60 to 60 ps. The upper and lower limits of the 95% confidence interval are less than two times the time resolution, while the standard deviation is less than 50 ps.

## 6. Discussion

According to the design scheme adopted in this article, the error of the delay generated by the delay generator should be less than two times the time resolution, and the 95% confidence intervals of the experimental data are indeed consistent with the expectation. With regards to experimental data, sometimes, there are some data with large errors, and the main reason is that the delay compensation scheme adopted in this article is rough. In the correction process of delay chain 1, a mapping table is established. The calculation related to the correction uses integers, which leads to a large error, the maximum of which is 54 ps (the delay time generated by one CARRY4 unit). In the correction process of delay chain 2, the input data of delay chain 2 is changed in this article. Similarly, the calculation related to the correction uses integers, which also leads to a large error, and the maximum is 54 ps. In this article, the inherent delay of the delay generator is calibrated, and the inherent delay also has error, but the error is small. Therefore, the maximum error of the experimental data is about 108 ps (the delay time generated by two CARRY4 units). The 95% confidence interval of experimental data can well reflect the error range of experimental data. 

In general, time accuracy refers to the standard deviation of the error between the generated delay and the preset delay. The standard deviation of experimental data is in the range of 30~40 ps, which is less than 50 ps. The purpose of the delay compensation scheme proposed in this article is to make the working state of the theoretical model of the delay generator designed in this article closer to the actual circuit. In addition, the delay generator is only a theoretical model and does not consider the error caused by some environmental factors, such as chip voltage fluctuation and temperature change.

In the follow-up research work, it is necessary to further research the characteristics of the CARRY4 unit and subdivide it into multiple delay units for use, so as to further improve the time resolution. The main development direction of the delay generator in the future is to improve the time resolution, reduce the resource occupancy rate, and reduce the nonlinearity of the carry delay chain. As the time resolution of the delay generator continues to improve, the delay scheme will gradually reach the physical limit of the hardware, and further improvements in the time resolution will be very difficult. Meanwhile, the improvement of time resolution will also be accompanied by the increase in the resource occupancy rate. It is necessary to reduce the resource occupancy rate under the condition of ensuring the performance of the delay generator. When the time resolution is high, the nonlinearity of the carry delay chain will have a great impact on the time accuracy, so improving the linearity of the carry delay chain will significantly improve the time accuracy. 

## 7. Conclusions

**Figure 22:**
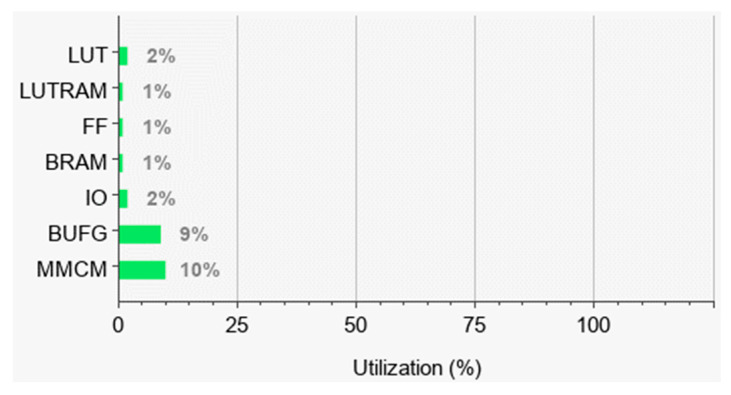
The resource occupancy rate of the FPGA.

The delay generator proposed in this article adopts the carry delay chain and achieves a high time resolution and a wide delay range through the combination of rough delay and precise delay. At the same time, a delay compensation scheme is proposed, which makes the working state of the theoretical model of the delay generator closer to the actual circuit. The biggest feature of this delay generator is its low resource occupancy rate and simple design scheme, and its resource occupancy rate is shown in Figure 22.

Compared with the existing delay generator, its time accuracy, delay range, and other performance indices are not different. Additionally, the biggest difference lies in its real-time requirement, so its design scheme is very different from the existing delay generator. Because it needs to wait for the external trigger signal to work, the corresponding calculation needs to be carried out in the delay process, which will lead to high hardware complexity. In fact, the same design scheme can also be implemented in ASIC, but FPGA has higher flexibility in verifying algorithms at a lower cost. Circuit design based on FPGA is a general design method. 

The biggest limitation of the method proposed in this article is that it is very dependent on the logic unit of FPGA. The time resolution of the delay generator directly depends on the delay time generated by the CARRY4 unit. With the continuous improvement of the time resolution, the method will gradually approach the physical limit of FPGA hardware, and it is difficult to further improve the time resolution. At the same time, there will be serious nonlinear phenomenon in the carry delay chain, which will greatly affect the time accuracy of the delay generator. 

The delay compensation scheme adopted in this article can make the working state of the theoretical model of the delay generator closer to the actual circuit, but the scheme is rough, which leads to the performance of the delay generator not being good. In fact, the delay compensation scheme adopted in this article will not be implemented in the actual circuit. In the actual circuit, the code density calibration method is generally used to obtain the actual parameters of each carry delay chain, and these parameters are used to calculate the delay time. Compared with the delay compensation scheme adopted in this article, the code density calibration method is more precise. In the actual circuit, multiple carry delay chains are generally used for measurement, and the length of each carry delay chain is shorter than that of the theoretical model. Obviously, the computation in the actual circuit is larger than that of the theoretical model.

Finally, it is verified, by simulation experiments, that the time resolution of the proposed delay generator is 54 ps, and the time accuracy is less than 50 ps. In fact, the time resolution achieved in this article is the lowest time resolution that can be achieved by the design scheme adopted in this article. If a CARRY4 unit is subdivided into multiple delay units, the time resolution can be further improved, but the resource occupancy rate of the FPGA will increase accordingly. As the number of delay units increases, the register array connected to the carry delay chain needs to use more registers, which increases the resource occupancy rate of the FPGA. Therefore, the trade-off between time resolution and resource occupancy rate of the proposed delay generator can be optimized by changing the tap extraction method of CARRY4 unit. At the same time, this article can also change the algorithm used in the encoding circuit of delay chain 1 to balance its encoding speed and its resource occupancy rate. 

## Figures and Tables

**Table 1:** Statistics of the first group of experimental data.

Preset Delay	Average Value	Confidence Interval	Standard Deviation
110.634 ns	110.630 ns	110.630 ± 0.066 ns	33.854 ps

**Table 2:** Statistics of the second group of experimental data.

Preset Delay	Average Value	Confidence Interval	Standard Deviation
110.688 ns	110.701 ns	110.701 ± 0.063 ns	32.116 ps

**Table 3:** Statistics of the error of the experimental data.

Preset Delay	Average Value	Confidence Interval	Standard Deviation
100~120 ns	3.982 ps	3.982 ± 69.871 ps	35.648 ps

## Data Availability

The data presented in this study are available on request from the corresponding author.
